# The diagnostic value of component-resolved diagnostics in peanut allergy in children attending a Regional Paediatric Allergology Clinic

**DOI:** 10.1186/s12887-016-0609-7

**Published:** 2016-06-02

**Authors:** Leonieke N. van Veen, Michiel Heron, Manou Batstra, Paul M. M. van Haard, Hans de Groot

**Affiliations:** Department of Paediatric Allergology, Reinier de Graaf Hospital, Delft, PO Box 5011, 2600 GA The Netherlands; Department of Medical Microbiology and Immunology, St. Elisabeth Hospital, Tilburg, The Netherlands; Medical Laboratories, Department of Immunology, Reinier de Graaf Hospital, Delft, The Netherlands; Medical Laboratories, Department of Clinical Chemistry, Reinier de Graaf Hospital, Delft, The Netherlands

**Keywords:** Anaphylaxis, Children, Double-blind, Placebo-controlled food challenge, Food allergy, Peanut allergy, Recombinant allergens

## Abstract

**Background:**

To date, diagnosing food allergies in children still presents a diagnostic dilemma, leading to uncertainty concerning the definite diagnosis of peanut allergy, as well as to the need for strict diets and the potential need for adrenalin auto-injectors. This uncertainty in particular is thought to contribute to a lower quality of life. In the diagnostic process double-blind food challenges are considered the gold standard, but they are time-consuming as well as potentially hazardous. Other diagnostic tests have been extensively studied and among these component-resolved diagnostics appeared to present a promising alternative: Ara h2, a peanut storage protein in previous studies showed to have a significant predictive value.

**Methods:**

Sixty-two out of 72 children, with suspected peanut allergy were analyzed using serum specific IgE and/or skin prick tests and specific IgE to several components of peanut (Ara h 1, 2, 3, 6, 8, 9). Subsequently, double-blind food challenges were performed. The correlation between the various diagnostic tests and the overall outcome of the double-blind food challenges were studied, in particular the severity of the reaction and the eliciting dose.

**Results:**

The double-blind provocation with peanut was positive in 33 children (53 %). There was no relationship between the eliciting dose and the severity of the reaction. A statistically significant relationship was found between the skin prick test, specific IgE directed to peanut, Ara h 1, Ara h 2 or Ara h 6, and the outcome of the food challenge test, in terms of positive or negative (*P* < .001). However, we did not find any relationship between sensitisation to peanut extract or the different allergen components and the severity of the reaction or the eliciting dose. There was no correlation between IgE directed to Ara h 3, Ara h 8, Ara h 9 and the clinical outcome of the food challenge.

**Conclusions:**

This study shows that component-resolved diagnostics is not superior to specific IgE to peanut extract or to skin prick testing. At present, it cannot replace double-blind placebo-controlled food challenges for determination of the eliciting dose or the severity of the peanut allergy in our patient group.

**Electronic supplementary material:**

The online version of this article (doi:10.1186/s12887-016-0609-7) contains supplementary material, which is available to authorized users.

## Background

Allergic reactions to peanut vary from mild localized or gastro-intestinal symptoms to severe generalized reactions affecting the respiratory and/or cardiovascular system [1]. This allergy can have a significant impact on the quality of life of children and their parents. Sometimes the case history is evident and the diagnosis of anaphylaxis caused by peanut is easily made. However, reactions to food tend to be difficult to categorize: the culprit food may remain unclear. Interpreting symptoms may be difficult, especially when symptoms are mostly subjective, or remain unrecognized due to unawareness by caretakers other than parents.

Defining sensitisation to peanut is the next step in the diagnostic work-up, but is insufficiently conclusive. A subsequent double-blind, placebo-controlled food challenge (DBPCFC) is considered to be the gold standard for diagnosing food allergy [2]. However, severe reactions can occur during DBPCFC and are reported in 10–12 % of patients challenged for peanut in previous studies [3–5]. Furthermore, these challenges are time-consuming and restricted to a few well-equipped pediatric hospitals with an allergological expert group. Performing a DBPCFC with peanut will usually confirm or exclude the diagnosis of peanut allergy, thus leading to either specific advice concerning exclusion of peanut from the diet, or to the possibility of safe introduction.

The main concerns of patients and/or parents of a child with food allergy are uncertainty and lack of information about the eliciting dose and the severity of the possibly allergic reaction. A recent Dutch study shows that adult patients, adolescents, and children all benefit from a negative DBPCFC outcome, but children benefit even if the outcome of the provocation is positive [6]. Other studies on parents’ perceptions of their child’s food allergy showed similarly reduced parental concerns and parental anxiety after food challenge, irrespective of the outcome of the challenge. It was hypothesized that these improvements in quality of life were caused by the fact that a definitive diagnosis of the food allergy provides a sense of security. This was more important than fears concerning the challenge procedure. In conclusion, a definite diagnosis after a positive or negative outcome improves quality of life [7, 8].

In the past decades component-resolved diagnostics (CRD) has become increasingly routine, with currently more than 130 allergenic molecules commercially available for in vitro specific IgE (sIgE) testing [9–11]. CRD is an approach used to map the allergen sensitisation of a patient at a molecular level, using purified natural or recombinant allergenic molecules instead of allergen extracts. It is suggested that CRD may be used to assess the risk of severe systemic reactions in food allergy, thereby reducing the need for DBPCFC in certain groups of allergic patients.

In this study we investigated the predictive value of component-resolved diagnostics for a clinical relevant allergy in a group of consecutive children suspected of a peanut allergy. Three objectives were of special interest:

- can we predict the positive or negative outcome of the DBPCFC with peanut by measuring the levels of specific IgE to different recombinant peanut allergens?
- can we predict the eliciting dose (ED) by using CRD?
- can we predict the severity of the allergic reaction occurring at the DBPCFC with peanut with this assay?
- Especially the second and third questions are of interest, because these are most important to parents and determine quality of life and their ability to cope with the allergy in the future.

## Methods

### Study design (Additional file 1)

In 2012 and 2013 consecutive patients attending our outpatient clinic were asked to participate in this prospective cohort study. These children were referred by general practitioners and pediatricians from surrounding hospitals in the western part of the Netherlands. Allergological examinations were performed in the Reinier de Graaf Hospital in Delft. All children underwent serological testing and/or skin testing on the first visit, provocations were done within approximately 3–4 months after the first visit. Data were collected and analysed in 2014.

Children were suspected of having a peanut allergy, because of a history of an allergic reaction in the past or sensitisation to peanut with no or unknown exposure. In the latter group the sensitisation was found by previous testing for various reasons. All children underwent an intensive case history regarding previous peanut ingestion or exposure. Previous reactions to peanut were reported by the parents of the child and classified according to the Sampson’s classification based on the most severe reaction observed, see Table 1 [1]. The presence of asthma, atopic dermatitis, allergic rhinitis, and other food allergies was determined in out-patient clinic consultations before DBPCFC.

**Table 1:** Grading of food-induced anaphylaxis according to severity of clinical symptoms [1]

Grade	Skin	GI tract	Respiratory tract	Cardiovascular	Neurological
1	Localized pruritus, flushing, urticaria, angioedema	Oral pruritis, mild lip swelling	None	None	None
2	Generalized pruritus, flushing, urticaria, angioedema	Any of the above, Nausea and or emesis x’s 1	Nasal congestion and/or sneezing	None	Change in activity level
3	Any of the above	Any of the above plus repetitive vomiting	Rhinorrhea, marked congestion, sensation of throat pruritus of tightness	Tachycardia	Change in activity level plus anxiety
4	Any of the above	Any of the above plus diarrhea	Any of the above, hoarseness, “barky”cough, difficulty swallowing, dyspnea, wheezing, cyanosis	Any of the above, dysrhythmia and or mild hypotension	“light headedness”, feeling of “pending doom”
5	Any of the above	Any of the above, loss of bowel control	Any of the above, respiratory arrest	Severe bradycardia and/or hypotension or cardiac arrest	Loss of consciousness

Subsequently, sensitisation was measured by skin prick test, sIgE determination and CRD (Immunocap ISAC). Within 3 months after the intake a DBPCFC with peanut was performed in the hospital. Only cases with all measurements available qualified for inclusion.

Data were obtained as part of regular patient care and used strictly anonymously, according to the code of conduct for medical research approved by the hospital’s Medical Ethical Committee. Parents gave their written informed consent before starting the provocation tests.

### Tests for sensitisation

Measurement of peanut-specific IgE (sIgE) was performed in all children using the 3gAllergy™ assay on an IMMULITE® 2000 XPi system (Siemens), according to the manufacturers instructions. Specific IgE titers were quantified (KU_A_/L) with a lower limit of normal of 0.35 KU_A_/L and an upper detection limit of 100 KU_A_/L as proposed by the manufacturer.

Specific IgE directed against peanut protein components Ara h 1, Ara h 2, Ara h 3, Ara h 6, Ara h 8 and Ara h 9 was determined using the Immuno Solid-phase Allergen Chip (ISAC) assay (Thermo Fisher Scientific, Uppsala, Sweden). Other major allergens from peanut were not available in this assay. Results were analyzed on a semiquantitative basis, and IgE values were presented in arbitrary units called ISAC standardized units (from 0.3 to 100 ISU). Values of >0.3 ISU were considered positive. The technique was performed following the manufacturer’s instructions [12, 13].

Skin prick tests (SPT) were performed within 3 months prior to DBPCFC using a commercial peanut extract (ALK-Abelló, Nieuwegein, the Netherlands). For positive and negative control histamine dihydrochloride, 10 mg/ml and glycerol diluent were used, respectively. Mean diameter of peanut wheal size in millimeters was calculated from the average of the largest wheal diameter plus largest wheal diameter perpendicular to this. SPT was also evaluated by using the HEP index (histamine equivalent pricktest), and considered positive when the wheal was 3 mm greater in diameter than the negative control and the HEP was more than 0.3 [14, 15].

### DBPCFC for peanut

Challenges were performed in a clinical setting equipped for resuscitation and monitoring of vital signs according to protocol. Only clinically stable children without recent infection, uncontrolled atopic disease or recent allergic reactions were eligible to start the challenge procedure. The DBPCFC protocol for peanut was described earlier by Vlieg & Flinterman et al. [16, 17]. The challenge was considered positive and terminated when objective symptoms occurred or when consistent subjective symptoms occurred on at least two subsequent doses. The eliciting dose (ED) was determined as the lowest dose of peanut protein (in mg) eliciting objective allergic reaction. Symptoms during challenge were graded after physical examination by one of the clinical experts and classified based on the most severe reaction observed in an organ system, according to the Sampson’s classification [1]. Severe food challenge outcome (FCO) was defined as a positive FCO with a severe respiratory and/or cardiovascular reaction of Sampson’s grade 3 or 4. These children were treated with epinephrine intramuscularly. Children with moderate or mild reactions were defined as having a reaction according to Sampson’s grade 2 and 1, respectively. These reactions were treated with oral antihistamines only.

The eliciting dose was graded as follows:

- Low dose, reacting on dose 1 (0.84 mg peanut protein) or dose 2 (1.6 mg peanut protein).
- Medium dose, reacting on dose 3 (8 mg peanut protein) or dose 4 (42 mg peanut protein).
- High dose, reacting on dose 5 (0.21 gram peanut protein, 4/10th of a whole peanut) or dose 6 (1.01 gram peanut protein, 2 whole peanuts).

### Statistical methods

Statistical analyses were performed using GraphPad Prism software package (version 6, GraphPad Software, Inc., San Diego, CA, USA). Comparisons were considered significant at a *P* value less than .05. The Wilcoxon-Mann-Whitney test was used to compare the allergic and tolerant groups and the Kruskal-Wallis test was used to compare the DBPCFC severity score categories and DBPCFC eliciting dose categories among the different measurements. The Fisher Exact Probability test was used to test the correlation between the DBPCFC severity score and the DBPCFC eliciting dose.

Statistical analyses of logistic regression and correlations were performed using StatGraphics Centurion XVII software (Version 17.1.08 for MS-Windows; Statpoint, Inc., Warrenton, VA, USA). To estimate odds ratios logistic regression with backward factor selection (*P*-to-enter .05; *P*-to-remove.05) was applied, with DBPCFC outcome as binominal response variable and numerical variables (Ara h components on ISAC, age) and categorical variables (gender) as factors. Non-parametric Spearman’s rank correlations were assessed between numerical factors before establishing the most adequate logistic regression model. Where applicable, a *P*-value < .05 was considered statistically significant at the 95 % confidence level.

## Results

A total of 72 consecutive children were included in this study. Because of anxiety and therefore lack of venapuncture and/or skin testing, 10 patients were excluded from the final analysis. The cohort predominantly consisted of boys (74 %) and children had a median age of 7.9 years (range 3–16 years). Applying the non-parametric Kruskal and Wallis test with the Bonferroni procedure (of pairwise comparisons between the average ranks of the 2 groups), none of the medians comparisons between the sexes were statistically significant at the 95 % confidence level for all recombinant peanut allergens tested.

In 37 children (60 %), the history revealed no known previous ingestion of peanut. Sensitisation to peanut and/or anxiety for allergic reactions had been reasons not to introduce peanut in the diet. In 17 children (27.4 %), peanut was ingested with subsequent mild to moderate allergic complaints; 8 children (13 %) had experienced a severe anaphylactic reaction to peanut in the past.

### Food challenge outcome

The DBPCFC with peanut was positive in 33 children (53 %). The severity of the reaction was grade 1 in 6 children, grade 2 in 22 children and severe (grade 3,4) in 5 children. The eliciting dose was low in 5 children, medium in 14 children and high in 14 children. There was no relationship between the eliciting dose and the severity of the reaction in the DBPCFC (data not shown).

**Fig. 1:**
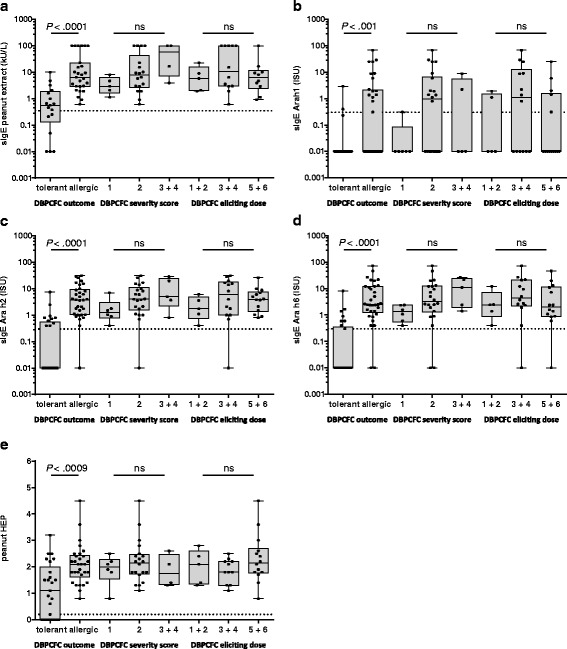
Relationship between sensitisation parameters and outcome of DBPCFC with peanut, in regard to positive/negative, severity score and eliciting dose. Sensitisation was established by means of sIgE to peanut (**a**), sIgE to Ara h 1 (**b**), sIgE to Ara h 2 (**c**), sIgE to Ara h 6 (**d**) and skin prick test with peanut (**e**). The data are presented as box and whiskers plots (25th to 75th percentiles, median, min en max values), including data points. The dashed lines represent the cut-off values

A statistically significant relationship was shown between the skin prick test, sIgE directed to peanut, Ara h 1, Ara h 2 or Ara h 6, and the outcome of the food challenge test, in terms of positive or negative (*P* < .001, Fig. 1). Moreover, the sensitivity of sIgE to peanut extract and the SPT with peanut were 100 %; while using sIgE to different peanut components several patients with a clinical relevant peanut allergy were missed.

We did not find a relationship between the sensitisations to peanut extract or the different allergen components and the severity of the reaction (categorised as severity 1, 2 or 3 & 4) or the eliciting dose (categorised as 1 & 2, 3 & 4, 5 & 6) (Figs. 1a–e). There was no correlation between IgE directed to Ara h 3, Ara h 8, Ara h 9 and the clinical outcome of the food challenge.

**Fig. 2:**
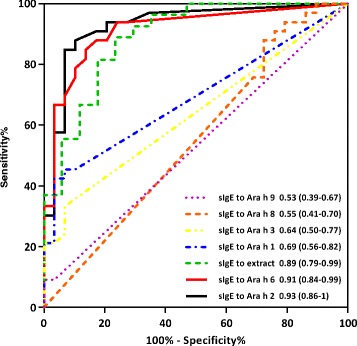
Receiver Operating Curve, data are presented for peanut components Ara h 1, 2, 3, 6, 8, 9 and peanut extract as area under the curve (95 % CI)

Analysis based on receiver operating characteristics (ROC curves) showed Ara h 2 and Ara h 6 to be the best discriminators between peanut allergy and tolerance (Fig. 2). Logistic regression analysis confirmed that Ara h 2 was the best predictor. A statistically significant model (adjusted percentage of deviance explained by model = 28 %), without being adequate yet (Likelihood Ratio test: *P* < .001) showed an odds ratio of 2.3 with a 95 % confidence interval ranging from 1.3 to 4.0, leaving only Ara h 2 as an explanatory variable. Unusual Residuals analyses suggested the presence of one outlier. After tentative removal of the outlier the model improved (adjusted percentage of deviance explained by model = 48 %), became adequate (Likelihood Ratio test: *P* = .32) and showed an odds ratio of 11.5 with a 95 % confidence interval ranging from 2.4 to 54.6, leaving only only Ara h 2 as an explanatory variable. A conclusion from the logistic regression analyses on this patients cohort is that we are 95 % confident that a rise in Ara h 2 (in International Standard Units, used in ISAC) increases the chance of a positive DBPCFC outcome with at least 30 % (or 140 % after omitting an outlier). In this study, Ara h 2 and Ara h 6 were highly correlated (non-parametric Spearman Rank Correlation: R2 = .81; i.e., .7 < |r| < .9, only complete data used; results not shown); moreover, on population level these measurands do not provide independent information since Ara h 2 and Ara h 6 are being considered very homologous.

## Discussion

Component-resolved diagnostics is becoming increasingly routine in the Netherlands. It may facilitate the management of allergic patients for the physician [9–11]. Knowledge of the exact allergenic molecules the patient is sensitised to can help predict the likelihood of mild versus severe systemic food reactions. Some allergens such as the storage proteins in peanuts and nuts have been shown to be associated with severe allergic reactions, while other allergens cause mild oral allergy or even positive allergy tests without clinical relevance. Furthermore, allergens that are stable to heat and digestion (e.g., Ara h 2 from peanut and Cor a 9 from hazelnut) are more likely to cause severe anaphylactic reactions, whereas heat and digestive labile molecules (e.g., Ara h 8 from peanut and Cor a 1 from hazelnut) are more likely to cause milder, local allergic reactions or no reactions at all [11, 18].

In a systematic review by Klemans et al. it was stated that sIgE directed to Ara h 2 was superior in diagnosing peanut allergy in case of a positive test result, compared to skin prick test and sIgE to whole peanut extract [19]. They reviewed 21 studies in pediatric cohorts and found a sensitivity rangeing 66–100 % with a specificity rangeing 0–95 % (food challenge as gold standard) for skin prick testing. For sIgE to peanut extract sensitivity ranged from 80 to 100 % with corresponding specificity from 0 to 63 %. For sIgE to Ara h 2 sensitivity ranged from 60 to 100 % with a specificity of 60–96 %. For sIgE directed to Ara h 1 or Ara h 3 diagnostic values varied widely; for the components Ara h 5, Ara h 8 and Ara h 9 the sensitivity values were low in all studies. They concluded that sIgE to Ara h 2 had the highest LR+, indicating that it was the best test to predict peanut allergy. The LR- was comparable to the SPT and sIgE to peanut extract [19].

In earlier study cohorts it was found that sIgE to storage proteins such as Ara h 1, 2, and 3 was associated with clear peanut allergic reactions; in contrast to Ara h 8, a PR-10 protein and Bet v 1 homologue, which is associated with milder or local allergic reactions like oral allergy symptoms [11, 18]. In southern Europe, the lipid transport protein (LTP) Ara h 9 is a prevalent sensitising allergen that may act as a marker of severe anaphylactic reactions to peanut [20]. It is still not known how prevalent Ara h 9 is in other geographical regions. Finally, patients with profiline or sensitisation to sugars (carbohydrate crossreactive determinants, CCD) in peanut alone, usually react with no or local oral symptoms and heated peanuts may be tolerated.

In this study we investigated the predictive value of the presence of specific IgE to recombinant peanut allergens (Ara h 1, 2, 3, 6, 8 and 9) for the outcome of a DBPCFC with peanut. More specific, the correlation between sIgE to different peanut components and the overall outcome of the DBPCFC, the eliciting dose of the DBPCFC and the severity of the reaction were studied.

### Can we predict the positive or negative outcome of the DBPCFC with peanut by measuring the levels of specific IgE to different recombinant peanut allergens?

We found a high negative predictive value for sIgE to peanut extract (100 %), similar to SPT and superior to the negative predictive value of specific IgE to individual peanut components. This means that with a negative test (sIgE to peanut or SPT), a food challenge will not be necessary anymore and peanut should be reintroduced into the diet. Based on the clinical assessment by the specialist experienced in allergology this introduction may be performed either at home (in which case we use a safe introduction scheme) or in the hospital [21].

The positive predictive value of all the diagnostic tests (sIgE, SPT, recombinant allergens) was low in our patient group. This means that in sensitised patients there is still a considerable chance that the oral provocation will turn out to be negative.

### Can we predict the eliciting dose and/or the severity of the allergic reaction by using CRD?

Most important, there was no correlation between the diagnostic tests performed and either the eliciting dose nor the severity of the allergic reaction during the food provocation. Hence DBPCFC will therefore be necessary in the future to determine this important information for the patients and their parents.

Our results are comparable with a recent study by Blumchen et al. [22]. They stated that a modified food challenge procedure in 63 children with peanut allergy with doses of peanut scheduled 2 h apart, showed a better reflection of the real-life thresholds for peanut allergy. They found a mild-to-moderate inverse correlation of the markers for sensitisation (sIgE to peanut, sIgE to Ara h 2 and SPT) with the eliciting dose at challenge. But, similar to our results, there was no correlation between the biological markers tested and the grade of severity of the most severe objective allergic reaction at challenge.

Bégin et al. investigated whether early CRD at time of peanut allergy diagnosis in children would provide information in addition to standard sIgE to better predict future evolution of the disease [23]. They have shown, Ara h 1, Ara h 2 and Ara h 3 to be the dominant peanut allergens in this population of very young children (median age, 14 months). For the follow-up of a median of 12 years, CRD with major peanut allergens did not provide additional independent predictive value for the persistence of peanut allergy.

### Study limitations

These data were obtained in a group of children with either a suspicion of food allergy based on sensitisation only or a peanut allergic reaction in the past. This being the major reason for referral to our practice. To confirm or rule out a clinical reactivity to peanut we routinely perform a DBPC food challenge. We were interested to study the applicability of CRD in this group of children. Other limitations are the size of the group, the specific region (western part of the Netherlands), and the fact that we can give recommendation only on peanut allergy. Finally, we excluded children who recently experienced a clear clinical reaction to peanut. In this group a DBPC food challenge will not be necessary to confirm the diagnosis. Because these patients were not included, it could have biased this study. In daily practice these patients are not willing to undergo a provocation and we intended to test the recombinant allergens on patient groups with unknown clinical reactivity, as seen in many pediatric clinics.

## Conclusion

This study shows that component-resolved diagnostics is not superior to sIgE to peanut extract or to skin prick testing to diagnose peanut allergy. At present, it cannot replace double-blind placebo-controlled food challenges for determination of the eliciting dose or the severity of the peanut allergy in our patient group. Further studies with larger numbers of patients and other regions are necessary to investigate the generalizability of these results in the case of peanut allergy.

## Abbreviations

CRD, component-resolved diagnostics; DBPCFC, double-blind placebo-controlled food challenge; ED, eliciting dose; FCO, food challenge outcome; ISAC, Immuno-Solid phase Allergen Chip; sIgE, specific IgE; SPT, skin prick test.

## Additional file

Additional file 1: The STARD 2015 list. (DOC 93 kb)
