# Supplementary material for: The diagnostic value of component-resolved diagnostics in peanut allergy in children attending a Regional Paediatric Allergology Clinic
Source: BMC Pediatr. 2016 Jun 2;16:74. doi: 10.1186/s12887-016-0609-7 (PMC4891901; doi:10.1186/s12887-016-0609-7)
Supplement: Additional file 1: — The STARD 2015 list. (DOC 93 kb) [file 12887_2016_609_MOESM1_ESM.doc]

**The STARD 2015 list**

| Section and topic | No | Item | Page |
| --- | --- | --- | --- |
| Title or abstract | | |  |
|  | **1** | Identification as a study of diagnostic accuracy using at least one measure of accuracy (such as sensitivity, specificity, predictive values, or AUC) | 2 |
| Abstract | | |  |
|  | **2** | Structured summary of study design, methods, results, and conclusions (for specific guidance, see STARD for Abstracts) | 2-3 |
| Introduction | | |  |
|  | **3** | Scientific and clinical background, including the intended use and clinical role of the index test | 3-5 |
|  | **4** | Study objectives and hypotheses | 5 |
| Methods | | |  |
| Study design | **5** | Whether data collection was planned before the index test and reference standard were performed (prospective study) or after (retrospective study) | 6 |
| Participants | **6** | Eligibility criteria | 6 |
|  | **7** | On what basis potentially eligible participants were identified (such as symptoms, results from previous tests, inclusion in registry) | 6 |
|  | **8** | Where and when potentially eligible participants were identified (setting, location, and dates) | 6 |
|  | **9** | Whether participants formed a consecutive, random, or convenience series | 6 |
| Test methods | **10a** | Index test, in sufficient detail to allow replication | 7 |
|  | **10b** | Reference standard, in sufficient detail to allow replication | 7 |
|  | **11** | Rationale for choosing the reference standard (if alternatives exist) | 7-8 |
|  | **12a** | Definition of and rationale for test positivity cut-offs or result categories of the index test, distinguishing pre-specified from exploratory | 8 |
|  | **12b** | Definition of and rationale for test positivity cut-offs or result categories of the reference standard, distinguishing pre-specified from exploratory | 8 |
|  | **13a** | Whether clinical information and reference standard results were available to the performers or readers of the index test | 8 |
|  | **13b** | Whether clinical information and index test results were available to the assessors of the reference standard | n.a. |
| Analysis | **14** | Methods for estimating or comparing measures of diagnostic accuracy | 9 |
|  | **15** | How indeterminate index test or reference standard results were handled | 9 |
|  | **16** | How missing data on the index test and reference standard were handled | 9 |
|  | **17** | Any analyses of variability in diagnostic accuracy, distinguishing pre-specified from exploratory | 10-11 |
|  | **18** | Intended sample size and how it was determined | n.a. |
| Results | | |  |
| Participants | **19** | Flow of participants, using a diagram | n.a. |
|  | **20** | Baseline demographic and clinical characteristics of participants | 9 |
|  | **21a** | Distribution of severity of disease in those with the target condition | 9-10 |
|  | **21b** | Distribution of alternative diagnoses in those without the target condition | n.a. |
|  | **22** | Time interval and any clinical interventions between index test and reference standard | n.a. |
| Test results | **23** | Cross tabulation of the index test results (or their distribution) by the results of the reference standard | n.a. |
|  | **24** | Estimates of diagnostic accuracy and their precision (such as 95% confidence intervals) | 10-11 |
|  | **25** | Any adverse events from performing the index test or the reference standard | n.a. |
| Discussion | | |  |
|  | **26** | Study limitations, including sources of potential bias, statistical uncertainty, and generalisability | 15 |
|  | **27** | Implications for practice, including the intended use and clinical role of the index test | 15 |
| Other information | | |  |
|  | **28** | Registration number and name of registry | n.a. |
|  | **29** | Where the full study protocol can be accessed | n.a. |
|  | **30** | Sources of funding and other support; role of funders | 17 |
